# How Education Shapes Indigenous Health Inequalities in the USA and Mexico

**DOI:** 10.1007/s40615-024-01922-4

**Published:** 2024-02-27

**Authors:** Gabriela León-Pérez, Elyas Bakhtiari

**Affiliations:** 1 Department of Sociology, Virginia Commonwealth University, Richmond, VA USA; 2 Department of Sociology, William and Mary, Williamsburg, VA USA

**Keywords:** Indigenous health, Cross-national research, American Indian, Indigenous Mexicans, Health disparities

## Abstract

Indigenous peoples around the world face significant health disparities relative to the dominant groups in their countries, yet the magnitude and patterns of health disparities vary across countries. We use data from the National Health Interview Survey and Mexican Family Life Survey to examine the health of Indigenous peoples in Mexico and American Indians and Alaska Natives in the USA and to evaluate how they fare relative to the majority populations in their countries (non-Indigenous Mexicans and non-Hispanic Whites, respectively). We assess disparities in self-rated health and activity limitations, with a focus on how Indigenous health disparities intersect with educational gradients in health. Regression analyses reveal three primary findings. First, Indigenous health disparities are larger in the USA than in Mexico. Second, differences in educational attainment account for most of the differences between Indigenous and non-Indigenous populations in Mexico, but less than half in the USA. Third, in both countries, health is moderated by educational attainment such that between-group disparities are largest at the highest levels of education. However, for Indigenous Mexicans there is a “cross-over” in which Indigenous Mexicans report better health at the lowest level of education. Overall, this study finds a weak relationship between education and Indigenous health, and raises the question about the validity of using traditional measures of SES in Indigenous contexts.

**Supplementary Information:**

The online version contains supplementary material available at 10.1007/s40615-024-01922-4.

## Introduction

The association between socioeconomic status (SES) and health typically follows a social gradient whereby those at the higher and lower levels of the SES hierarchy experience the best and poorest health outcomes, respectively [1–3]. These differences not only exist at the high and low ends but also are evident at any point along the continuum, such that individuals will tend to have better health than those with lower SES [4, 5].

However, there is growing evidence that the positive health effects of SES are systematically smaller for racial and ethnic minorities [6–9]. This pattern of diminishing returns leads to larger ethnic or racial disparities at higher levels of SES given that the smaller health gains accumulate at the highest levels of the socioeconomic ladder [10–12]. While there is consistent evidence of diminishing returns among racial and ethnic minorities, particularly African Americans, it is still unclear if this same pattern is observed among Indigenous populations (in the USA and abroad) who collectively experience social, political, and economic marginalization, discrimination, and disproportionately lower SES. Indeed, as Shepherd and colleagues [13] argue, “scant attention has been paid to the potential moderating effect of Indigenous status on the SES-health relationship.”

Indigenous peoples’ health is “behind everyone, everywhere” [14], yet their degree of disadvantage may vary depending on the specific national context. Cross-national comparisons are valuable for assessing how Indigenous peoples are faring in countries with distinct social, political, economic, and health care contexts. In addition, comparing Indigenous health disparities across countries may shed light on variability in the traditional domains of social determinants of health.

This study examines the health of Indigenous peoples in Mexico and the USA and evaluates how they fare relative to the majority populations in their countries (non-Indigenous Mexicans and non-Hispanic Whites, respectively). Using data from the Mexican Family Life Survey [15] and the National Health Interview Survey [16], we assess disparities in self-rated health and activity limitations with a focus on how Indigenous health disparities intersect with educational gradients in health. Specifically, we are interested in assessing whether education plays a role in closing the gap in health between Indigenous populations and majority groups or, alternatively, if the diminished returns pattern is present in one or both countries. The unique socio-historical circumstances and profound marginalization that characterize the Indigenous context may constrain the health benefits that are usually gained from improved SES.

Most existing cross-national studies of Indigenous health disparities have focused on comparisons between the USA, Canada, Australia, and/or New Zealand [17–19], all of which are developed nations that share similar colonial histories [20, 21]. To our knowledge, this is the first study that uses national survey data to simultaneously study Indigenous health disparities in the USA and Mexico. There are substantial differences in social, structural, health care, and living conditions across these two countries which might contribute to cross-national differences in Indigenous health outcomes and disparities. Contemporary differences in the experiences of Indigenous peoples in the USA and Mexico go back to the distinct histories of British and Spanish colonial practices and post-Independence policies relating to Indigenous matters. While the USA was driven by the ideology of White supremacy and the disappearance of the Indigenous past, Mexico emphasized its Indigenous heritage and Indigenous-centered origin stories [22]. Despite these differences, Indigenous culture was seen in Mexico and the USA as “backward” and inconsistent with modernization, and both countries aimed at incorporating the Indigenous populations through processes of assimilation into the mainstream cultures. Today, Indigenous peoples in Mexico and the USA share the historical struggles related to structural racism and the legacies of colonization and racial/ethnic nation-building projects. Moreover, the neoliberal ideologies in both countries have fostered policies that undermine Indigenous collectivist values, and created structures of disadvantage and a new form of paternalism akin to those produced by colonization [23]. Taken together, past and present social, political, and economic policies and practices have shaped the way that US and Mexican institutions relate to Indigenous peoples, ultimately influencing Indigenous health outcomes.

## Background

### Indigenous Health and SES

Indigenous peoples around the world face significant health disparities relative to the dominant groups (usually considered the benchmark population) in their countries. Despite improvements over the last century, Indigenous groups continue to have lower life expectancies; higher rates of infant, child, and maternal mortality; and higher rates of infectious and chronic diseases, alcohol and drug abuse, and depression [24–27]. Although socioeconomic disadvantages help explain part of the health disparities that Indigenous groups experience relative to their non-Indigenous counterparts [28], disparities persist even after considering socioeconomic differences. Thus, some scholars argue that the idea of SES as a fundamental cause of health has limited applicability for Indigenous populations and that there are grounds for questioning the existence of a presumed linear relationship between SES and Indigenous health [29, 30].

The diminished returns hypothesis posits that greater SES does not confer equal advantages to racial and ethnic minorities as it does to non-marginalized groups. That is, as the socioeconomic standing of racial and ethnic minorities increases, they do not experience the same improvements in health. Because they do not enjoy similar returns in health for higher SES achievement, the racial gap in health tends to be largest as the highest levels of SES. Between-group differences are often smaller or negligible at the lowest levels of SES. Research in support of this hypothesis has focused primarily on Black-White disparities in the USA across a variety of health outcomes, primarily in self-rated health [12, 31–33], but also other such as allostatic load [34], mental health [8], infant birthweight [35], and obesity [36].

One challenge to comparing Indigenous health status across levels of SES is the overrepresentation of Indigenous peoples in the lower levels, which weakens statistical power as we move up the SES continuum and could possibly obscure the SES-health relationship [13]. Nevertheless, there is theoretical support for expecting to find a pattern of diminished returns in the relationship between SES and Indigenous health. First, discrimination and systemic racism may inhibit the benefits that normally accumulate by moving up the SES ladder. In practice, this is often observed, for example, in high levels of chronic stress caused by experiences of discrimination [37–39], differential treatment in the health care system [40–42], and differences in environmental conditions and access to resources due to residential segregation [43, 44]. Racism and discrimination are key factors underlying the relatively flatter SES-health gradients of African Americans in the USA [45, 46]. Second, the systematic marginalization and intergenerational trauma that Indigenous peoples have been historically subjected to can place them at a disadvantage from the earliest stages of life (indeed, from the womb), thus hindering the health benefits at every level of SES [29, 47]. Third, there may be factors other than traditional indicators of SES that play a more substantive role in shaping Indigenous health outcomes, such as involvement with their Indigenous culture, family support, strong communities, and connection to the land [48–50].

There are few empirical assessments of the moderating effect of SES in the context of health disparities experienced by Indigenous peoples, much less from a cross-national perspective. Shepherd and colleagues [13] reviewed the literature on Indigenous Australian health and found some evidence of a positive social gradient in mortality, kidney disease, diabetes, and smoking status, but no evidence in other health outcomes. They posit that these mixed findings may reflect the variety of health and SES measures, as well as the diverse Indigenous population groups and analytic techniques used across studies. In the USA, Ross et al. [51] tested the diminished returns hypothesis using a population-based sample of California births and found that Native American women had higher risk of diabetes and shorter gestational length than White women. Higher SES (measured using education and insurance status) attenuated the risk for White women but not for Native American women. Similarly, Nguyen, Moser, and Chou [9] examined the social gradient across racial/ethnic groups in California and found that increases in SES were not associated with better health among Black Americans and American Indians, particularly when they used education as the SES indicator. To our knowledge, there are no prior studies examining the SES-health gradient among Indigenous Mexicans.

### Indigenous Health and SES Across National Contexts

Not only does SES have a differential impact across population groups within a country, but there is also evidence of cross-national variations in the shape of SES-health gradients. For example, low SES leads to higher degrees of disadvantage in the USA than in Canada, the United Kingdom, and Germany [52]. Research suggests that the shape of the SES-health gradient, as well patterns of ethnic and racial health disparities, is related to a country’s social, cultural, and economic institutions that distribute health-related resources [53–55]. Hence, it is likely that the SES-health gradient for Indigenous populations may also be different across national contexts. In some countries, Indigenous groups have been afforded a range of linguistic, cultural, and territorial rights; in others, they have little or no recognition by the government and experience active suppression of their culture, language, and political aspirations [56]. These contextual differences shape social determinants of health and may lead to different SES-Indigenous health patterns. Alternatively, it could also be possible to find similar cross-national patterns of diminished returns which would suggest that, despite distinct national contexts, there is a generalizable pattern of how ethno-racial stratification interacts with SES to shape Indigenous health.

Prior international studies have demonstrated that it is not appropriate to make direct comparisons between Indigenous populations living in different countries given differences in data collection methods and in the measurement of Indigenous status [57, 58]. In addition to these methodological problems, direct comparisons are difficult because each country defines indigeneity differently and the social, political, economic, racial, and health care structures vary widely across countries. Nevertheless, cross-national studies that assess the health outcomes for Indigenous peoples relative to benchmark populations can provide important public health insights and inform the development of policy and service responses [24]. As Kumanyika [59] argues, while policy solutions to health disparities must be developed within the particular contexts in which they arise, such policies “can be informed by an appreciation of the commonalities and differences in how they occur and are remedied in different societies.”

### Indigenous Peoples in Mexico and the USA

There are 11.8 million Indigenous peoples living in Mexico and they account for 9.4% of the national population [60]. In the USA, there are 3.7 million individuals (1.1% of the population) who identify as American Indian and Alaska Natives (AIAN) [61]. The size of the US Indigenous population goes up to 9.7 million or 2.9% if we also consider individuals who identify as AIAN in combination with another race [62].

The history and institutional legacies of colonization and racism in both countries continue to perpetuate interpersonal and systemic forms of discrimination and marginalization. In the USA, the poverty rate among AIANs (25.9%) is double the national rate (12.8%) and also higher than the rates for all other racial/ethnic groups (Black, 21.7%; Hispanic, 17.7%; Asian/Native Hawaiian and Pacific Islander, 10.2%; White, 9.5%) [63]. A substantially larger disparity is observed in Mexico where 69.5% of Indigenous peoples live in poverty compared to 39% of their non-Indigenous counterparts [64]. In both countries, discrimination based on Indigenous background continues to shape social, residential, educational, and employment opportunities [37, 65].

In terms of educational attainment, Indigenous Mexicans have completed an average of 6 years of schooling and approximately 23% are illiterate; comparatively, the non-Indigenous Mexican population has completed an average of 9 years of education and 5.5% are illiterate [66]. While Indigenous Mexicans are lagging in education, there have been substantial improvements over the past twenty years. In 1990, the average educational attainment for Indigenous peoples was only 2 years and 41% were illiterate [66]. In the USA, approximately 80% of AIANs have completed high school and 15% have bachelor’s degrees or higher; in comparison, 93% of White Americans are high school graduates and 37% have bachelor’s degrees or higher [67].

AIANs and Indigenous Mexicans face structural disadvantages and exhibit poorer health outcomes than the benchmark groups in their countries across a range of health indicators, including higher mortality rates, lower life expectancy, and higher rates of communicable diseases [24–27]. This was especially evident during the COVID-19 pandemic. During the first 2 years of the pandemic, the Indigenous mortality rate in Mexico was 52–68% higher than among the non-Indigenous population [68–70]. In the USA, AIANs had the worst outcomes of all racial/ethnic groups. Age-adjusted data from the Centers for Disease Control and Prevention (CDC) reveal that, compared to non-Hispanic Whites, the rate of COVID-19 infection among AIANs was 2.2 times higher, their hospitalization rate was 2.7 times higher, and their death rate was 2.9 times higher [71].

There is one notable body of research that compared the health of Pima, an Indigenous group that is spread across Arizona and the Mexican states of Sonora and Chihuahua. Decades-long research has identified a consistently high prevalence of obesity and type 2 diabetes among the US Pima [72]. Remarkably, cross-national studies reveal that Mexican Pima are healthier than their US counterparts and have similar health outcomes to non-Indigenous Mexicans. For instance, Schulz et al. [73] found that the prevalence of diabetes among Pima in Mexico is significantly lower than US Pima (6.9% vs. 38%) and slightly higher than that of non-Indigenous Mexicans (2.9%). Similar patterns were observed in obesity, whereby rates were similar among Mexican Pima (men 7%; women 20%) and non-Indigenous Mexicans (men 9%, women 27%), but substantially higher among US Pima (men 64%; women 75%) [73]. Other studies using the same data found that US Pima also have higher insulin resistance (a diabetes risk factor [74]) and lower levels of physical activity [75].

## Current Study

The current study seeks to investigate how the SES-health gradient operates among Indigenous peoples and the role of SES in closing (or not) the gap between Indigenous and non-Indigenous peoples. To this end, we test interactions between Indigenous status and education to examine if Indigenous health disparities are consistent across different levels of educational attainment. Second, we seek to gain insights into whether the shape of the SES-health gradient for Indigenous peoples differs across countries with diverse social, political, and economic realities. We achieve this goal by using cross-national health data to investigate the shape of the gradients for Indigenous peoples living in the USA and Mexico. Examining the relationship between SES and indigeneity across countries provides an opportunity to advance scholarship on Indigenous health by identifying cross-national similarities and differences in health disparities and, more generally, to advance theory on social determinants of health by identifying variations in the shape of the SES-health gradient for ethnic-racial minorities across national contexts.

## Methods

### Data Source

We used data from the National Health Interview Survey (NHIS) and the Mexican Family Life Survey (MxFLS) to analyze Indigenous population health in Mexico and the USA. The NHIS is an annual survey administered by the National Center for Health Statistics to a representative sample of the noninstitutionalized population of the USA [16]. For this study, we pooled annual NHIS survey data from 2000 to 2018 to obtain a sufficient Indigenous sample size. The MxFLS is a longitudinal, nationally representative study of the well-being of individuals and families living in Mexico [15]. The MXFLS sample was collected using a probabilistic, stratified, and multi-staged cluster design, and is representative at the national, regional, and urban–rural regional levels. To maximize comparability with the NHIS, we used MxFLS-3 data collected between 2009 and 2012. Both surveys were limited to respondents 25 and over to capture the age range likely to have completed formal education.

### Variables

Our first outcome of interest is self-rated health. Prior research confirms that this is an adequate measure of health among Indigenous populations and that it is associated with mobility, physical health, and emotional well-being [76–78]. The NHIS and MxFLS rely on different response scales related to self-rated health. The response categories in each survey differ in wording (e.g., “regular” in the MxFLS roughly corresponds to “fair” in the NHIS) and the MxFLS question provides a balanced scale (very bad, bad, regular, good, very good), whereas the NHIS version is unbalanced (poor, fair, good, very good, excellent). Previous comparisons suggest the distributions of responses across different self-rated health measures are not always directly comparable, however there is evidence that the different questions measure the same latent variable and a linear coding scheme is preferred to maximize equivalence across question forms [79, 80]. For this reason, we rely on a linear coding for both surveys, where 1 represents the best level of health in the response scale, and 5 represents the worst level of health. In supplemental analyses, provided in the accompanying appendix, we tested an alternative version in which we dichotomized responses in both surveys and found similar results.

The second outcome of interest is activity limitations. Questions assessing functional limitations were more reliable and directly comparable across surveys. Therefore, our measure of functional activity limitations was re-coded as a binary variable in both datasets, with 1 indicating a reported activity limitation.

The primary independent variable similarly relies on different questions to identify Indigenous individuals in each survey. The MxFLS asked the following question to capture indigeneity: “Do you consider yourself part of an Indigenous group?” Respondents were coded as 1 = Indigenous if they answered yes. This operationalization is consistent with the criteria used by the Mexican census to identify Indigenous populations (INEGI, 2010). The NHIS does not specifically ask about Indigenous background, but respondents who selected “American Indian or Alaska Native” in response to the race question were coded as Indigenous. In order to capture the relative social hierarchies in each country, the reference category in Mexico was non-Indigenous Mexicans, whereas in the USA, it was the non-Hispanic White population.^1^ Thirteen percent of MxFLS-3 respondents and 1.1% of NHIS respondents self-identified as Indigenous.

The second key independent variable is educational attainment. This SES indicator presents multiple advantages, including that is stable over time (i.e., does not decrease across the lifespan), is less susceptible to reverse causation (i.e., when the health outcome precedes and results in the exposure), and the social gains from education are fairly consistent across countries [83]. We use different categories across the two surveys to reflect the distinct educational systems within each country. In the NHIS data, categories include the following: less than high school, high school, some college, college degree, and graduate degree. In the MxFLS, categories are: no schooling, elementary school (grades 1–6), middle school (grades 7–9), high school (grades 10–12), and college or more. The categories representing the lowest level of education in each country are used as the reference.

### Analytic Strategy

We estimate ordinary least squares regressions for self-rated health and logistic regressions for activity limitations. Analyses involved a series of nested models that subsequently: (1) assess relative health inequalities, (2) control for educational attainment, (3) introduce an interaction between education and Indigenous background, and (4) add controls for age (continuous), sex (1 = female), health insurance status (1 = has health insurance), and recent visits for health care (1 = health care visit in last four weeks). Rows with missing values were deleted using listwise deletion.^2^

Our analysis does not compare rates of health status across countries. We are specifically interested in relative disparities between Indigenous peoples and the dominant group in their respective countries, rather than cross-country comparisons between Indigenous peoples. Indeed, the latter is problematic given that conceptualizations of Indigenous identity, health systems, social and economic circumstances, and racial and social structures vary between countries [58]. Thus, each survey is analyzed independently to assess relative health inequalities in each context. Although we offer some comparative interpretation of the results, our method entails parallel national analyses rather than direct cross-national comparison.

## Results

Table 1 presents descriptive statistics for the two survey samples. Across both the MxFLS and NHIS, the mean self-rated health score for Indigenous populations was higher than the mean for the non-Indigenous comparison group, suggesting worse self-rated health for Indigenous groups in the USA (2.64 vs. 2.26) and Mexico (2.60 vs. 2.51). When comparing activity limitations, there is a similar Indigenous disadvantage in the USA, where 27% of the Indigenous sample report limitations compared to 17% of non-Hispanic Whites. However, there is no difference in unadjusted levels of activity limitations in Mexico, where 10–11% of both Indigenous and non-Indigenous groups report activity limitations.

**Table 1:** Descriptive statistics for National Health Interview Survey (NHIS) and Mexican Family Life Survey (MxFLS) by indigenous status

NHIS	Non-Hispanic White	Indigenous
Self-rated health (mean)	2.26	2.64
Activity limitations (%)	0.17	0.27
Education (%)		
Less than high school	0.08	0.18
High school	0.30	0.34
Some college	0.29	0.32
College degree	0.21	0.11
Graduate degree	0.12	0.06
Female (%)	0.52	0.53
Age (years)	51.52	47.61
Has health insurance (%)	0.90	0.71
Recent medical visit (%)	0.20	0.21
*n*	*685,510*	*7,618*
MxFLS	Non-Indigenous	Indigenous
Self-rated health (mean)	2.51	2.60
Activity limitations (%)	0.10	0.11
Education (%)		
No schooling	0.10	0.25
Elementary school	0.40	0.48
Middle school	0.26	0.16
High school	0.12	0.05
College or more	0.12	0.06
Female (%)	0.57	0.54
Age (years)	45.6	48.9
Has health insurance (%)	0.65	0.55
Recent medical visit (%)	0.16	0.14
*n*	*14,334*	*2,181*

In the NHIS, Indigenous respondents are those who selected “American Indian or Alaska Native” in response to the race question; non-Indigenous respondents represent non-Hispanic White individuals. In the MxFLS, Indigenous respondents are those who indicated they are part of an Indigenous group; non-Indigenous respondents are those who do not consider themselves part of an Indigenous group

The descriptive results also highlight the different structure of educational hierarchies within the two countries. Completing high school is much less common in Mexico, for both Indigenous and non-Indigenous populations. Nearly 83% of Indigenous respondents in the NHIS had graduated high school, compared to only 11% in the MxFLS. However, the relative inequalities within each survey are more comparable. For instance, despite the very different distributions across educational attainment levels, the ratio of non-Indigenous to Indigenous college attainment is greater in the USA than in Mexico.

Tables 2 and 3 present regression results for the Mexican and U.S. samples, respectively. Our results focus on three primary findings. First, there is more consistent evidence of population-level health inequalities based on Indigenous background in the USA. Although the Indigenous populations of both countries are more likely to report poor self-rated health than the majority population in the unadjusted models (model 1a in Tables 2 and 3), the size of the relative inequality is larger in the USA (*b* = 0.38, *p* < 0.001) than in Mexico (*b* = 0.10, *p* < 0.001). In addition, there is a similar inequality when looking at activity limitations in the NHIS data (*b* = 0.56, *p* < 0.001), but there are no significant differences in rates of activity limitations by Indigenous status in the MXFLS (*b* = 0.06, *p* = 0.55) (model 2a in Tables 2 and 3).

**Table 2:** Regression of self-rated health and activity limitations by educational attainment and Indigenous status, Mexico (MxFLS)

	Self-rated health				Activity limitations			
	Model 1a	Model 1b	Model 1c	Model 1d	Model 2a	Model 2b	Model 2c	Model 2d
Indigenous	0.10***	0.00	− 0.14***	− 0.13***	0.06	− 0.04	− 0.21	− 0.18
	(0.07 to 0.13)	(− 0.03 to 0.03)	(− 0.21 to − 0.07)	(− 0.20 to − 0.06)	(− 0.09 to 0.20)	(− 0.19 to 0.11)	(− 0.51 to 0.08)	(− 0.49 to 0.12)
Education (ref: no schooling)								
Elementary school		− 0.08***	− 0.12***	− 0.02		− 0.33***	− 0.39***	− 0.39***
		(− 0.12 to − 0.05)	(− 0.16 to − 0.08)	(− 0.06 to 0.02)		(− 0.48 to − 0.18)	(− 0.55 to − 0.22)	(− 0.57 to − 0.21)
Middle school		− 0.34***	− 0.39***	− 0.18***		− 0.45***	− 0.50***	− 0.44***
		(− 0.37 to − 0.30)	(− 0.43 to − 0.34)	(− 0.22 to − 0.13)		(− 0.62 to − 0.29)	(− 0.68 to − 0.32)	(− 0.65 to − 0.23)
High school		− 0.50***	− 0.55***	− 0.33***		− 0.60***	− 0.63***	− 0.59***
		(− 0.54 to − 0.46)	(− 0.60 to − 0.50)	(− 0.38 to − 0.28)		(− 0.82 to − 0.39)	(− 0.86 to − 0.41)	(− 0.84 to − 0.34)
College or more		− 0.60***	− 0.64***	− 0.44***		− 0.59***	− 0.64***	− 0.57***
		(− 0.64 to − 0.55)	(− 0.69 to − 0.60)	(− 0.49 to − 0.39)		(− 0.81 to − 0.38)	(− 0.86 to − 0.41)	(− 0.82 to − 0.32)
Education × Indigenous status interactions								
Elementary school			0.12**	0.13**			0.25	0.32
			(0.04 to 0.20)	(0.05 to 0.21)			(− 0.11 to 0.62)	(− 0.06 to 0.69)
Middle school			0.29***	0.30***			0.23	0.27
			(0.18 to 0.39)	(0.20 to 0.40)			(− 0.25 to 0.70)	(− 0.22 to 0.75)
High school			0.32***	0.32***			− 0.08	− 0.16
			(0.17 to 0.47)	(0.17 to 0.47)			(− 1.00 to 0.70)	(− 1.10 to 0.64)
College or more			0.28***	0.26***			0.17	0.09
			(0.14 to 0.42)	(0.12 to 0.40)			(− 0.62 to 0.86)	(− 0.70 to 0.81)
Intercept	2.51***	2.76***	2.80***	2.28***	− 2.18***	− 1.80***	− 1.76***	− 2.10***
	(2.49 to 2.52)	(2.73 to 2.79)	(2.76 to 2.83)	(2.22 to 2.34)				
Num. obs	16,515	16,515	16,515	16,515	16,515	16,515	16,515	16,515

^***^p < 0.001, ^**^p < 0.01, ^*^p < 0.05

Estimates include 95% confidence intervals in parentheses. Self-rated health ranges from 1 to 5 where 5 represents the worst health. Activity limitations is a dichotomous variable where 1 represents having at least one activity limitation. Results for self-rated health are based on linear regression models, and results for activity limitations are based on logistic regression models. Models 1d and 2d include controls for age, sex, health insurance status, and recent health care visit

**Table 3:** Regression of self-rated health and activity limitations by educational attainment and Indigenous status, United States (NHIS)

	Self-rated health				Activity limitations			
	Model 1a	Model 1b	Model 1c	Model 1d	Model 2a	Model 2b	Model 2c	Model 2d
Indigenous	0.38***	0.23***	0.15***	0.22***	0.56***	0.33***	0.09	0.40***
	(0.34 to 0.42)	(0.20 to 0.27)	(0.08 to 0.22)	(0.15 to 0.29)	(0.49 to 0.63)	(0.25 to 0.42)	(− 0.08 to 0.25)	(0.23 to 0.58)
Education (ref: less than high school)								
High school		− 0.56***	− 0.56***	− 0.45***		− 0.94***	− 0.95***	− 0.78***
		(− 0.57 to − 0.54)	(− 0.57 to − 0.54)	(− 0.46 to − 0.44)		(− 0.97 to − 0.92)	(− 0.97 to − 0.93)	(− 0.81 to − 0.75)
Some college		− 0.78***	− 0.78***	− 0.62***		− 1.20***	− 1.21***	− 0.93***
		(− 0.79 to − 0.76)	(− 0.79 to − 0.76)	(− 0.64 to − 0.61)		(− 1.22 to − 1.17)	(− 1.23 to − 1.18)	(− 0.96 to − 0.90)
College degree		− 1.12***	− 1.12***	− 0.92***		− 1.93***	− 1.94***	− 1.59***
		(− 1.13 to − 1.11)	(− 1.14 to − 1.11)	(− 0.93 to − 0.90)		(− 1.96 to − 1.90)	(− 1.97 to − 1.91)	(− 1.62 to − 1.55)
Graduate degree		− 1.19***	− 1.19***	− 1.04***		− 1.93***	− 1.94***	− 1.74***
		(− 1.21 to − 1.18)	(− 1.21 to − 1.18)	(− 1.06 to − 1.03)		(− 1.97 to − 1.90)	(− 1.97 to − 1.90)	(− 1.78 to − 1.71)
Education × Indigenous status interactions								
High school			0.03	0.03			0.18	0.24*
			(− 0.05 to 0.12)	(− 0.05 to 0.12)			(− 0.00 to 0.36)	(0.04 to 0.43)
Some college			0.16***	0.10*			0.41***	0.27**
			(0.07 to 0.26)	(0.01 to 0.18)			(0.22 to 0.60)	(0.07 to 0.46)
College degree			0.14*	0.07			0.58***	0.40**
			(0.02 to 0.26)	(− 0.05 to 0.18)			(0.29 to 0.86)	(0.11 to 0.69)
Graduate degree			0.08	0.03			0.52**	0.36
			(− 0.05 to 0.22)	(− 0.10 to 0.16)			(0.17 to 0.87)	(− 0.01 to 0.72)
Intercept	2.26***	3.03***	3.03***	2.24***	− 1.58***	− 0.40***	− 0.40***	− 3.10***
	(2.26 to 2.27)	(3.02 to 3.04)	(3.02 to 3.05)	(2.22 to 2.26)	(− 1.59 to − 1.57)	(− 0.43 to − 0.38)	(− 0.42 to − 0.38)	(− 3.15 to − 3.05)
Num. obs	690,481	690,481	690,481	690,481	690,481	690,481	690,481	690,481

^***^p < 0.001, ^**^p < 0.01, ^*^p < 0.05

Estimates include 95% confidence intervals in parentheses. Self-rated health ranges from 1 to 5 where 5 represents the worst health. Activity limitations is a dichotomous variable where 1 represents having at least one activity limitation. Results for self-rated health are based on linear regression models, and results for activity limitations are based on logistic regression models. Models 1d and 2d include controls for age, sex, health insurance status, and recent health care visit

Second, educational attainment appears to attenuate population-level health inequalities in Mexico to a greater degree than in the USA. In our second set of regression models (models 1b and 2b in Tables 2 and 3), adding educational attainment as a measure of socioeconomic status accounts for less than half of the self-rated health and activity limitation inequalities between AIAN and non-Hispanic White populations in the USA. A similar model for the Mexican population more fully accounts for the initial inequalities between Indigenous and non-Indigenous Mexicans, as initial differences in self-rated health are no longer statistically significant (*b* = 0.00, *p* = 0.89).

**Fig. 1:**
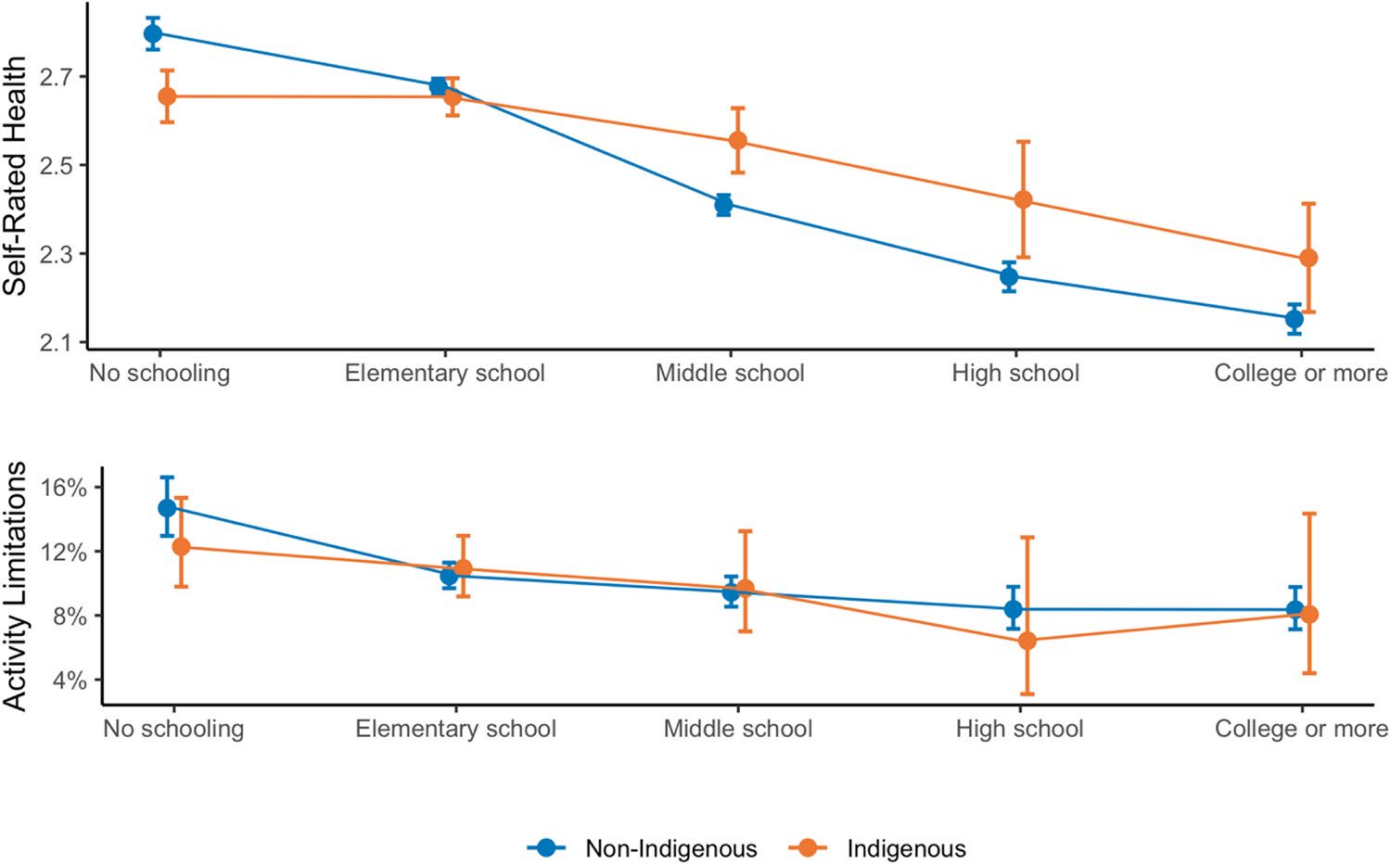
Education gradients in health for Indigenous and non-Indigenous populations in Mexico. *Note*: Data comes from the Mexican Family Life Survey (MxFLS-3). Figures depict predicted self-rated health scores (top) and probability of activity limitations (bottom) based on models with interactions between educational attainment and a categorical variable comparing Indigenous and non-Indigenous status. Self-rated health ranges from 1 to 5 where 5 represents the worst health. Activity limitations is a dichotomous variable where 1 represents having at least one activity limitation

**Fig. 2:**
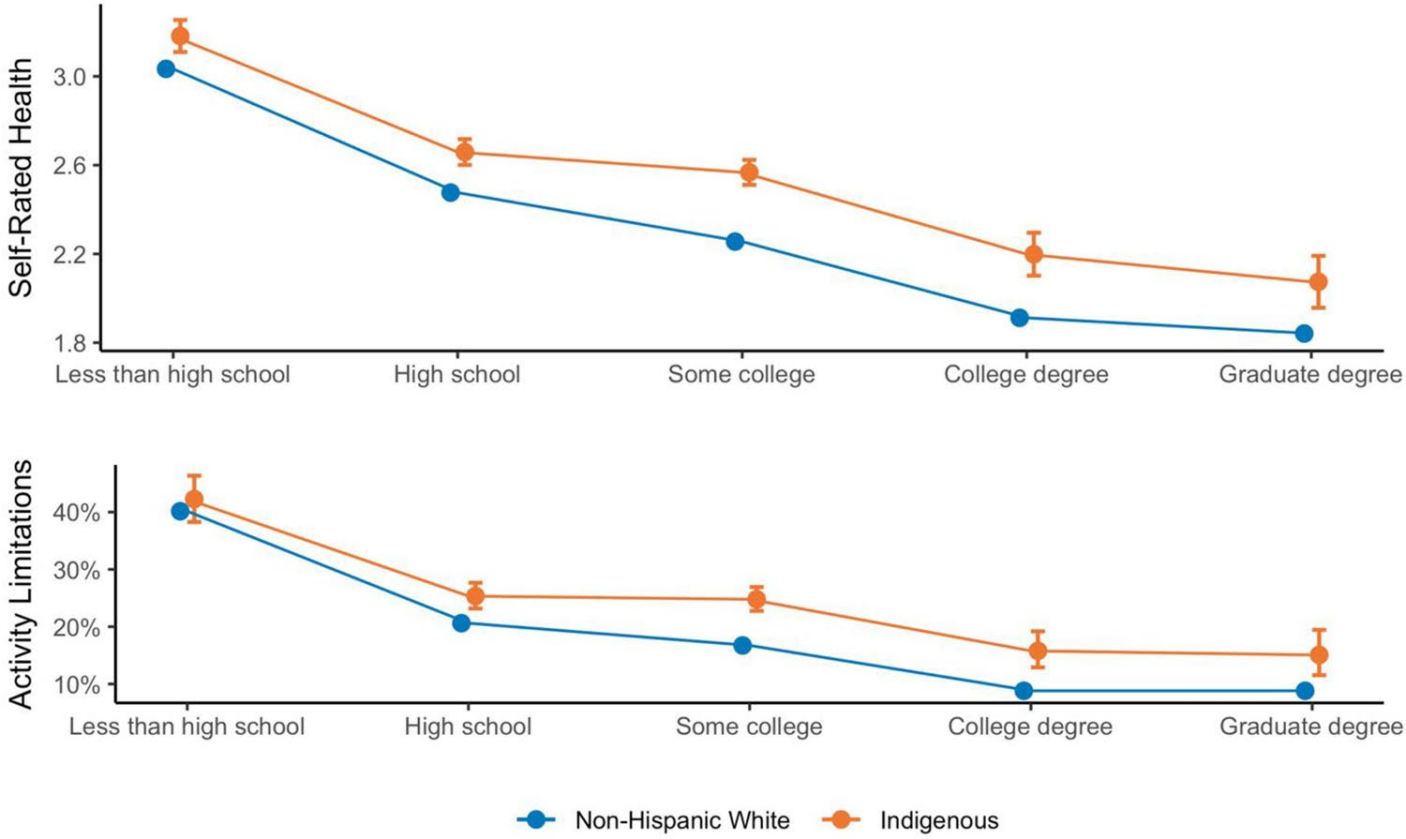
Education gradients in health for Indigenous and non-Hispanic White populations in the USA. *Note*: Data comes from the National Health Interview Survey, 2000-2018. Figures depict predicted self-rated health scores (top) and probability of activity limitations (bottom) based on models with interactions between educational attainment and a categorical variable comparing Indigenous and non-Indigenous status. Self-rated health ranges from 1 to 5 where 5 represents the worst health. Activity limitations is a dichotomous variable where 1 represents having at least one activity limitation

Third, there is evidence in both countries of a “diminished returns” pattern, in which Indigenous populations have different SES-health gradients compared to the benchmark populations, as indicated by an interaction between educational attainment and Indigenous background. The positive coefficients at higher levels of education for self-rated health across both surveys and activity limitations in the NHIS suggest the health improvements associated with higher levels of education (indicated by the negative coefficients for the education variable) are smaller for Indigenous populations relative to their non-Indigenous counterparts. Figures 1 and 2 illustrate the interaction from Models 1c and 2c in each survey. Looking at predicted probabilities across both surveys, trend lines based on the interactions show improvements in health with educational attainment across both measures and populations. However, the education improvements in self-rated health appear greater for non-Indigenous populations at higher education levels, resulting in relative inequalities being larger at the highest levels of educational attainment. This is similar to interactions with socioeconomic status seen for other marginalized groups [12, 33, 34].

However, the predicted probabilities reveal different trends in the relative gradients across contexts. In the USA, there are relatively small between-group inequalities at low levels of education, but the non-Hispanic White population has larger health gains as SES increases, so that relative Indigenous disadvantages are higher among college-educated populations up to the point of graduate-level degrees. Although the data in Mexico suggests similar diverging gradients between Indigenous and non-Indigenous populations, the Indigenous sample reports better self-rated health than non-Indigenous Mexicans at the lowest levels of educational attainment. The interaction crossover and unexpectedly good health at the lowest levels of educational attainment may partly explain the smaller inequalities observed when only comparing between populations without an interaction effect.

## Discussion

The current study presented an examination of the social gradient for Indigenous peoples in the USA and Mexico. It contributes to the literature by illustrating the unique patterns of the Indigenous social gradient in two countries where Indigenous groups are marginalized but within distinct social contexts. Using data collected in the USA and Mexico, we examined the interaction between Indigenous status and educational attainment. We also sought to uncover whether the shape of the social gradient is consistent for Indigenous peoples living in countries with differing social, political, and economic realities.

Our analyses revealed three main findings. First, relative Indigenous health disparities are larger in the USA than in Mexico. Second, differences in educational attainment account for most of the differences between Indigenous and non-Indigenous populations in Mexico, but less than half in the USA. Third, we found evidence of a diminished socioeconomic health gradient for Indigenous peoples in both countries, whereby self-rated health inequalities were larger at higher levels of educational attainment. Below we discuss these findings and their implications for scholarship and policy.

In the USA, consistent with the diminished returns hypothesis, increases in SES do not confer equal advantages to AIANs. Not only did AIANs experience poorer health across all education levels, but the Indigenous gap was largest at the highest levels of education. Although our study did not examine mechanisms for this pattern, recent literature suggests that stress responses, environmental exposures, structural racism, and historical trauma warrant further investigation in the context of SES gradients [29, 37, 44, 47]. Another possible explanation for our findings related to AIAN disparities could be that many tribes lack recognition by the federal government due to technical oversights and disagreements over historical treaties [84, 85]. The US government officially recognizes 574 tribes, but there are approximately 400 tribes (with tens of thousands of members) that do not enjoy federal recognition. This lack of recognition can have significant health consequences because it prevents these communities from accessing resources, such as those provided by the Indian Health Service. During the COVID-19 pandemic, unrecognized tribes were unable to access federally-funded testing and vaccination initiatives, such as the $31 billion infusion of COVID-19 relief into tribal communities [86]. As a result, members of unrecognized tribes had to depend on neighboring tribes for access to testing and vaccines. In this context, efforts to reduce AIAN disparities must also address systemic barriers that prevent Indigenous peoples from accessing services from the Indian Health Service and government funds earmarked for American Indian communities. Future studies should systematically investigate if there are disparities within AIANs based on tribal recognition.

The findings for Mexico are inconclusive. The interaction model for activity limitations (illustrated in Fig. 1) indicated that there are no significant differences between Indigenous and non-Indigenous Mexicans across levels of education. In the case of self-rated health, there is a “cross-over” in which Indigenous Mexicans report better health than the non-Indigenous at the lowest level of education. While this result was unexpected, Farmer and Ferraro [12] had a similar finding in their study of Black-White differences in the SES-health gradient. For self-rated health, they found that Black Americans with the lowest levels of education reported better health than their White counterparts, but then as education increased the self-rated health of White individuals improved substantially and the gap widened. Nguyen et al. [9] found a curvilinear social gradient among Latinos and Asian Americans, whereby those with the lowest SES had healthier outcomes, health worsened as SES increased, and then improved again at the highest levels of SES. In our study, as education increased past elementary school, there was a larger improvement in the self-rated health for non-Indigenous than for Indigenous Mexicans. However, the gap closed at the highest levels of education. The latter result should be interpreted with caution given the small number of Indigenous Mexicans with college education in the MxFLS sample.

Our finding that Indigenous peoples in Mexico experience smaller health disparities than AIANs in the US is consistent with research that found that the health of Mexican Indigenous peoples from the Pima tribe is significantly better than that of US Pima and similar to the health of non-Indigenous Mexicans [73]. Other studies have found improvements in the health of Mexican Indigenous children and adults in recent years, as well as reductions in Indigenous health disparities [87–90]. Some scholars suggest that this might be related to increased access to health care through the creation and expansion of universal health care in Mexico [89, 91]. However, despite these improvements, significant disparities still exist in a variety of health outcomes, including immunizations, stunting, infant mortality, health care utilization, and maternal health outcomes [42, 87, 92].

This research is not without limitations. First, there are 68 Indigenous groups in Mexico and 574 federally recognized Indian tribes in the USA. The heterogeneity (in terms of language, cultural practices, region) may produce variation in health outcomes within Indigenous populations. It is, however, difficult to disaggregate data into these more specific Indigenous groups because individuals’ group membership is rarely collected and the small sizes of some of these groups might make it difficult to conduct meaningful comparative analyses. Another limitation is that of statistical power. Given that AIANs represent such a small proportion of the overall US population, existing health surveys designed to gather population prevalence data often do not have enough statistical power to elicit information about this group. Indeed, we found that several studies that examined racial/ethnic differences in the social gradient excluded AIANs from their analyses [32, 36, 93]. In New Zealand, there have been efforts to oversample Māori populations in national health surveys [94] and we suggest that a similar strategy should be undertaken in the USA. Moreover, such sampling efforts can extend beyond national-level surveys. Due to the lack of cross-national samples of Indigenous populations, most of the variables in our study were not directly comparable across the two countries, resulting in parallel national analyses rather than an explicit cross-national comparison.

Despite the limitations, this study underscores the value of taking a comparative approach to understand patterns of Indigenous health disparities in different societies. Taken together, our results suggest that, at a national level, Indigenous peoples in the USA are more disadvantaged in health terms than Indigenous peoples in Mexico. This cross-national variation in SES-health disparities suggests that social gradients cannot be attributed only to overrepresentation in lower levels of SES, but rather they are socially produced [52].

A weak relationship between education and health among Indigenous peoples has implications for policy. Because of the long-standing patterns of health deprivation experienced by Indigenous peoples, improving their health outcomes is a central focus of public health policies in both countries. While government investments in education are generally beneficial, our results suggest that these are unlikely to lead to substantial improvements in Indigenous population health or reductions in Indigenous health disparities. Rather, what is likely needed are policies that address structural factors related to Indigenous inequities in a variety of areas, including access to health care, employment, housing, water, and healthy food.

Walter and Saggers [30] argue that “the social, political, and economic consequences of being an Indigenous person […] add a dimension that cannot simply be plugged into existing mainstream models” of the social determinants of health. Our paper provides support to this statement by showing that education—a SES indicator that is often described as “the most important modifiable social determinant of health” [95]—offers only a partial explanation of Indigenous health disparities. To the extent that SES describes an unequal distribution of resources, it may overlap with issues of colonialism, racism, and policy [96].

To conclude, this study raises the question about the validity of using traditional measures of SES in Indigenous contexts. Income, education, and employment may represent different constructs for Indigenous peoples (indeed, this may also be the case for health constructs) across different social and national contexts. It could be, as Altman [97] suggests, that social status for these communities may be more related to knowledge or to control rather than accumulation of material resources. Another problem is that measures like employment and income depend on the formal labor market and on cash incomes. However, many Indigenous peoples are frequently employed in the informal economy or in traditional livelihood activities; thus, they often have irregular income streams and many are paid in kind rather than cash [98] Existing measures of economic status are grounded on a market-oriented perspective of capitalistic society rather than on Indigenous peoples’ reality and their understanding of economic and social status [97]. This suggests the need to redefine our existing notions of SES in order to have a better understanding of its complex relationship with Indigenous health [13]. In line with decolonial methodologies, this redefinition must be developed in conversation with Indigenous peoples in order to fully capture their own interpretations of social status, well-being, and health [99, 100].

## Supplementary information

ESM 1 (DOCX 322 KB)

## Data Availability

All data used in this study can be accessed through the National Health Interview Survey and Mexican Family Life Survey. Replication code is available from the authors.

## Footnotes

1 In the USA, society is structured along racial-ethnic lines and resources are unequally distributed across racial/ethnic groups. Within this racial structure, non-Hispanic whites are the dominant group and racial/ethnic minorities are at a disadvantage [81]. In Mexico, ethnic categorizations tend to distinguish between Indigenous vs. non-Indigenous individuals—that is, the two main socially recognized ethnic categories [82]. Social inequality is structured along those lines whereby non-Indigenous Mexicans have an advantaged social position.

2 See the accompanying appendix for additional information about the missing data structure in each survey.
