# Supplementary material for: How Education Shapes Indigenous Health Inequalities in the USA and Mexico
Source: J Racial Ethn Health Disparities. 2024 Feb 27;12(2):837–50. doi: 10.1007/s40615-024-01922-4 (PMC11914346; doi:10.1007/s40615-024-01922-4)
Supplement: Supplementary file 1 — (DOCX 322 KB) [file 40615_2024_1922_MOESM1_ESM.docx]

**How Education Shapes Indigenous Health Inequalities in the United States and Mexico**

Appendix

Table of Contents

[A. MxFLS Survey Information 2](#_Toc156469990)

[Figure A1. Co-occurrence of Missing Values in the Mexican Family Life Survey, Wave 3 2](#_Toc156469991)

[B. NHIS Survey Information 3](#_Toc156469992)

[Table B1. Imputed Regression of Self-Rated Health and Activity Limitations by Educational Attainment and Indigenous Status, United States (NHIS) 3](#_Toc156469993)

[C. Analysis with Dichotomized Self-Rated Health 5](#_Toc156469994)

[Table C1. Regression of Binary Coding of Poor Self-Rated Health and Indigenous Status, Mexico (MxFLS) 6](#_Toc156469995)

[Table C2. Regression of Binary Coding of Poor Self-Rated Health and Indigenous Status, United States (NHIS) 7](#_Toc156469996)

[Figure C1. Education Gradients in Poor Self-Rated Health Health for Indigenous and non-Indigenous Populations in the United States and Mexico 8](#_Toc156469997)

# A. MxFLS Survey Information

The MxFLS is a longitudinal, nationally representative study of the well-being of individuals and families living in Mexico. The MXFLS sample was collected using a probabilistic, stratified, and multi-staged cluster design, and is representative at the national, regional, and urban-rural regional levels.

The MxFLS subsample of variables used in this analysis was characterized by a sizeable co-occurrence of missing variables. Of the 1,097 rows containing a missing value, 85% were missing at least 7 or more variables, as depicted in Figure A1. This made multiple imputation of missing values infeasible, as many of these rows has little remaining information to use for imputation. List-wise deletion was used to analyze the complete cases, as described in the Methods section of the manuscript.

| Figure A1. Co-occurrence of Missing Values in the Mexican Family Life Survey, Wave 3 |
| --- |
| 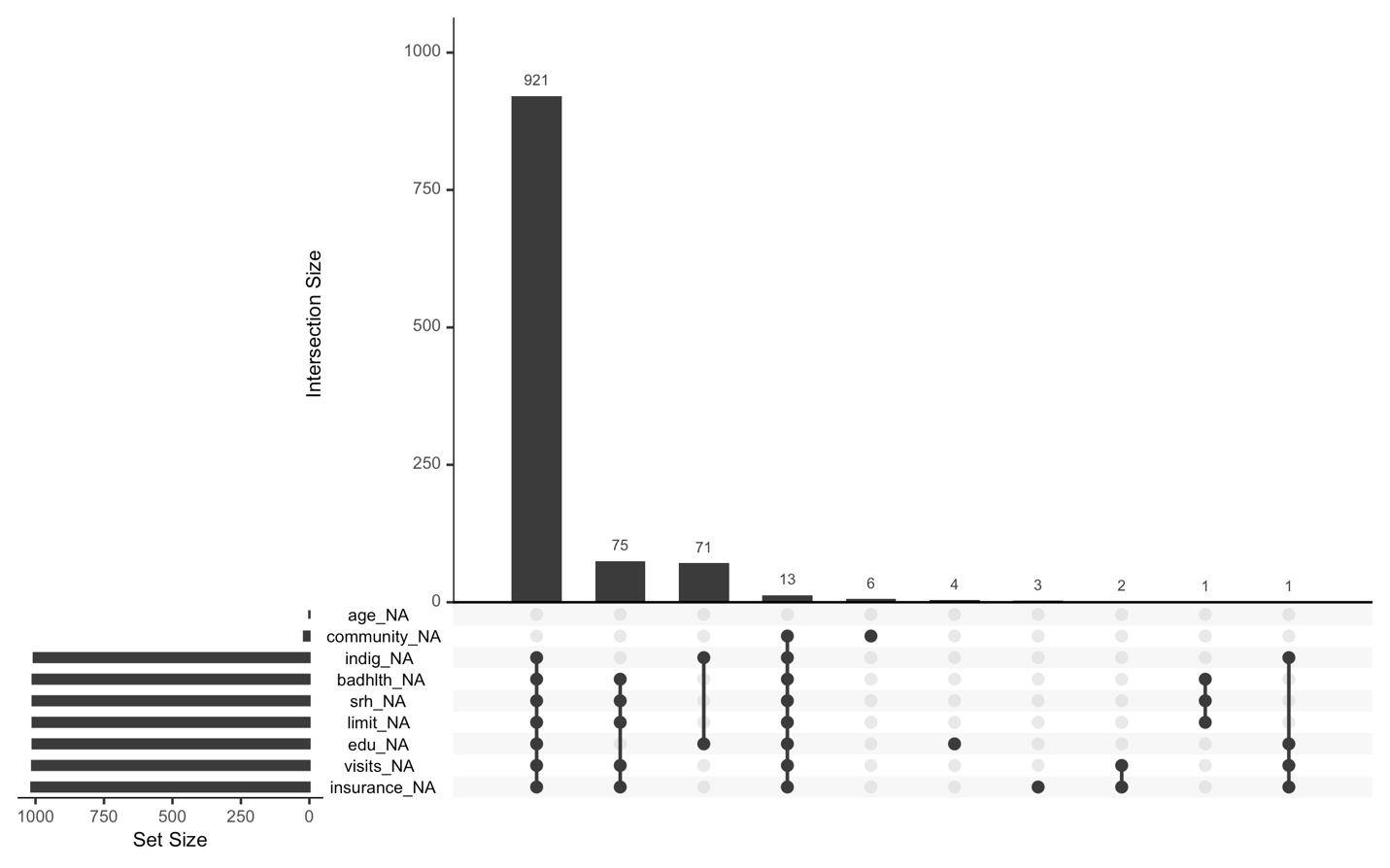 |
| *Note: Bars represent the number of rows with the combination of missing values indicated below.* |

# B. NHIS Survey Information

The NHIS is an annual survey administered by the National Center for Health Statistics to a representative sample of the noninstitutionalized population of the United States. For this study, we pooled annual NHIS survey data from 2000 to 2018 to obtain a sufficient Indigenous sample size.

Missing values were handled with list-wise deletion, to match the approach for handling the MxFLS (see Appendix A). However, as shown in Table B1, results from an alternative analysis using imputation to estimate missing values produced virtually identical results. Missing values in the NHIS were imputed using multivariate imputation by chained equations (mice) with a predictive mean matching method. Table B1 presents to pooled results of five imputed datasets, following the same analytic approach is the main manuscript.

Whereas weights were included with the NHIS survey to account for the survey design, the MxFLS dataset did not have a complete set of weights for the respondents in our sample.

| Table B1. Imputed Regression of Self-Rated Health and Activity Limitations by Educational Attainment and Indigenous Status, United States (NHIS) | | | | | | | | | | | | |  |
| --- | --- | --- | --- | --- | --- | --- | --- | --- | --- | --- | --- | --- | --- |
|  | Self-Rated Health | | | | Activity Limitations | | | | | | | |  |
|  | Model 1a | Model 1b | Model 1c | Model 1d | Model 2a | | Model 2b | | Model 2c | | Model 2d | | |
| **Indigenous** | 0.37 *** | 0.23 *** | 0.15 *** | 0.22 *** | 0.56 *** | | 0.33 *** | | 0.10 | | 0.42 *** | | |
|  | (0.34 - 0.41) | (0.19 - 0.26) | (0.08 - 0.22) | (0.15 - 0.29) | (0.49 - 0.63) | | (0.25 - 0.42) | | (-0.07 - 0.27) | | (0.24 - 0.59) | | |
| **Education (ref: Less than high school)** | | | | |  | | | | | | | |  |
| High school |  | -0.55 *** | -0.56 *** | -0.45 *** |  | | -0.94 *** | | -0.95 *** | | -0.78 *** | | |
|  |  | (-0.57 - -0.54) | (-0.57 - -0.54) | (-0.46 - -0.43) |  | | (-0.97 - -0.92) | | (-0.97 - -0.93) | | (-0.80 - -0.75) | | |
| Some college |  | -0.77 *** | -0.78 *** | -0.62 *** |  | | -1.20 *** | | -1.20 *** | | -0.93 *** | | |
|  |  | (-0.79 - -0.76) | (-0.79 - -0.76) | (-0.64 - -0.61) |  | | (-1.22 - -1.17) | | (-1.23 - -1.18) | | (-0.96 - -0.90) | | |
| College degree |  | -1.12 *** | -1.12 *** | -0.91 *** |  | | -1.93 *** | | -1.93 *** | | -1.58 *** | | |
|  |  | (-1.13 - -1.10) | (-1.13 - -1.11) | (-0.93 - -0.90) |  | | (-1.96 - -1.90) | | (-1.96 - -1.90) | | (-1.62 - -1.55) | | |
| Graduate degree |  | -1.19 *** | -1.19 *** | -1.04 *** |  | | -1.93 *** | | -1.93 *** | | -1.74 *** | | |
|  |  | (-1.20 - -1.17) | (-1.20 - -1.18) | (-1.05 - -1.02) |  | | (-1.96 - -1.90) | | (-1.97 - -1.90) | | (-1.78 - -1.71) | | |
| **Education × Indigenous Status Interactions** | | |  |  |  |  | |  | |  | |  |  |
| High school |  |  | 0.03 | 0.03 |  | |  | | 0.17 | | 0.22 * | | |
|  |  |  | (-0.05 - 0.12) | (-0.05 - 0.11) |  | |  | | (-0.01 - 0.35) | | (0.03 - 0.42) | | |
| Some college |  |  | 0.16 *** | 0.10 * |  | |  | | 0.40 *** | | 0.26 ** | | |
|  |  |  | (0.07 - 0.26) | (0.01 - 0.19) |  | |  | | (0.21 - 0.59) | | (0.07 - 0.46) | | |
| College degree |  |  | 0.13 * | 0.06 |  | |  | | 0.56 *** | | 0.38 ** | | |
|  |  |  | (0.01 - 0.25) | (-0.05 - 0.17) |  | |  | | (0.28 - 0.84) | | (0.09 - 0.67) | | |
| Graduate degree |  |  | 0.08 | 0.03 |  | |  | | 0.50 ** | | 0.32 | | |
|  |  |  | (-0.05 - 0.22) | (-0.10 - 0.16) |  | |  | | (0.13 - 0.85) | | (-0.04 - 0.70) | | |
| Intercept | 2.27 *** | 3.03 *** | 3.04 *** | 2.23 *** | -1.58 *** | | -0.40 *** | | -0.40 *** | | -3.13 *** | | |
|  | (2.26 - 2.27) | (3.02 - 3.05) | (3.02 - 3.05) | (2.21 - 2.25) | (-1.59 - -1.57) | | (-0.43 - -0.38) | | (-0.42 - -0.38) | | (-3.18 - -3.09) | | |
| Num. obs. | 714384 | 714384 | 714384 | 714384 | 714384 | | 714384 | | 714384 | | 714384 | | |
| Imputations | 5 | 5 | 5 | 5 | 5 | | 5 | | 5 | | 5 | | |

^***^p < 0.001, ^**^p < 0.01, ^*^p < 0.05

# C. Analysis with Dichotomized Self-Rated Health

The analysis of self-rated health in the main document is based on a 1-5 coding of self-rated health analyzed as a continuous scale using ordinary least squares linear regression. The following tables and figures present the results of an alternative analysis in which self-rated health was re-coded as a binary variable representing “fair or poor health” and analyzed using logistic regression.

Because the response scales differ across the two surveys, the binary coding differs across the two self-rated health questions. The NHIS response scale includes “poor,” “fair,” “good,” “very good,” and “excellent.” We recoded “poor” and “fair” as 1, and other responses as 0. The response categories differ in the MxFLS (i.e., very bad, bad, regular, good, very good), with “regular” roughly corresponding in meaning to “fair” in the NHIS. Thus, we recoded the MxFLS self-rated health question, with “very bad”, “bad”, and “regular” equal to 1.

| Table C1. Regression of Binary Coding of Poor Self-Rated Health and Indigenous Status, Mexico (MxFLS) | | | | |
| --- | --- | --- | --- | --- |
|  | Poor Self-Rated Health | | | |
|  | Model 1a | Model 1b | Model 1c | Model 1d |
| **Indigenous** | 0.290 *** | 0.052 | -0.350 *** | -0.337 ** |
|  | (0.047) | (0.049) | (0.103) | (0.106) |
| **Education (ref: No schooling)** | | | | |
| Elementary school |  | -0.037 | -0.130 * | 0.131 * |
|  |  | (0.053) | (0.062) | (0.065) |
| Middle school |  | -0.720 *** | -0.871 *** | -0.352 *** |
|  |  | (0.057) | (0.065) | (0.072) |
| High school |  | -1.207 *** | -1.355 *** | -0.823 *** |
|  |  | (0.068) | (0.075) | (0.083) |
| College or more |  | -1.509 *** | -1.664 *** | -1.184 *** |
|  |  | (0.071) | (0.078) | (0.084) |
| **Education × Indigenous Status Interactions** | | |  |  |
| Elementary school |  |  | 0.274 * | 0.313 * |
|  |  |  | (0.124) | (0.128) |
| Middle school |  |  | 0.842 *** | 0.906 *** |
|  |  |  | (0.153) | (0.157) |
| High school |  |  | 0.956 *** | 1.012 *** |
|  |  |  | (0.224) | (0.229) |
| College or more |  |  | 0.961 *** | 0.951 *** |
|  |  |  | (0.217) | (0.222) |
| Intercept | 0.057 *** | 0.581 *** | 0.694 *** | -0.644 *** |
|  | (0.017) | (0.049) | (0.056) | (0.097) |
| Num. obs. | 16515 | 16515 | 16515 | 16515 |

^***^p < 0.001, ^**^p < 0.01, ^*^p < 0.05

| Table C2. Regression of Binary Coding of Poor Self-Rated Health and Indigenous Status, United States (NHIS) | | | | |
| --- | --- | --- | --- | --- |
|  | Poor Self-Rated Health | | | |
|  | Model 1a | Model 1b | Model 1c | Model 1d |
| **Indigenous** | 0.765 *** | 0.458 *** | 0.181 | 0.354 *** |
|  | (0.061) | (0.066) | (0.103) | (0.103) |
| **Education (ref: Less than high school)** | | | | |
| High school |  | -1.151 *** | -1.158 *** | -0.987 *** |
|  |  | (0.020) | (0.020) | (0.022) |
| Some college |  | -1.526 *** | -1.539 *** | -1.307 *** |
|  |  | (0.022) | (0.023) | (0.025) |
| College degree |  | -2.422 *** | -2.431 *** | -2.108 *** |
|  |  | (0.032) | (0.032) | (0.035) |
| Graduate degree |  | -2.610 *** | -2.626 *** | -2.416 *** |
|  |  | (0.043) | (0.044) | (0.045) |
| **Education × Indigenous Status Interactions** | | |  |  |
| High school |  |  | 0.293 * | 0.320 * |
|  |  |  | (0.130) | (0.133) |
| Some college |  |  | 0.572 *** | 0.450 ** |
|  |  |  | (0.148) | (0.149) |
| College degree |  |  | 0.524 | 0.391 |
|  |  |  | (0.283) | (0.285) |
| Graduate degree |  |  | 1.100 *** | 1.003 ** |
|  |  |  | (0.320) | (0.321) |
| -3.399 *** | -2.047 *** | -2.039 *** | -3.745 *** | -3.399 *** |
| (0.009) | (0.017) | (0.017) | (0.040) | (0.009) |
| Num. obs. | 690481 | 690481 | 690481 | 690481 |

^***^p < 0.001, ^**^p < 0.01, ^*^p < 0.05

| Figure C1. Education Gradients in Poor Self-Rated Health Health for Indigenous and non-Indigenous Populations in the United States and Mexico |
| --- |
| 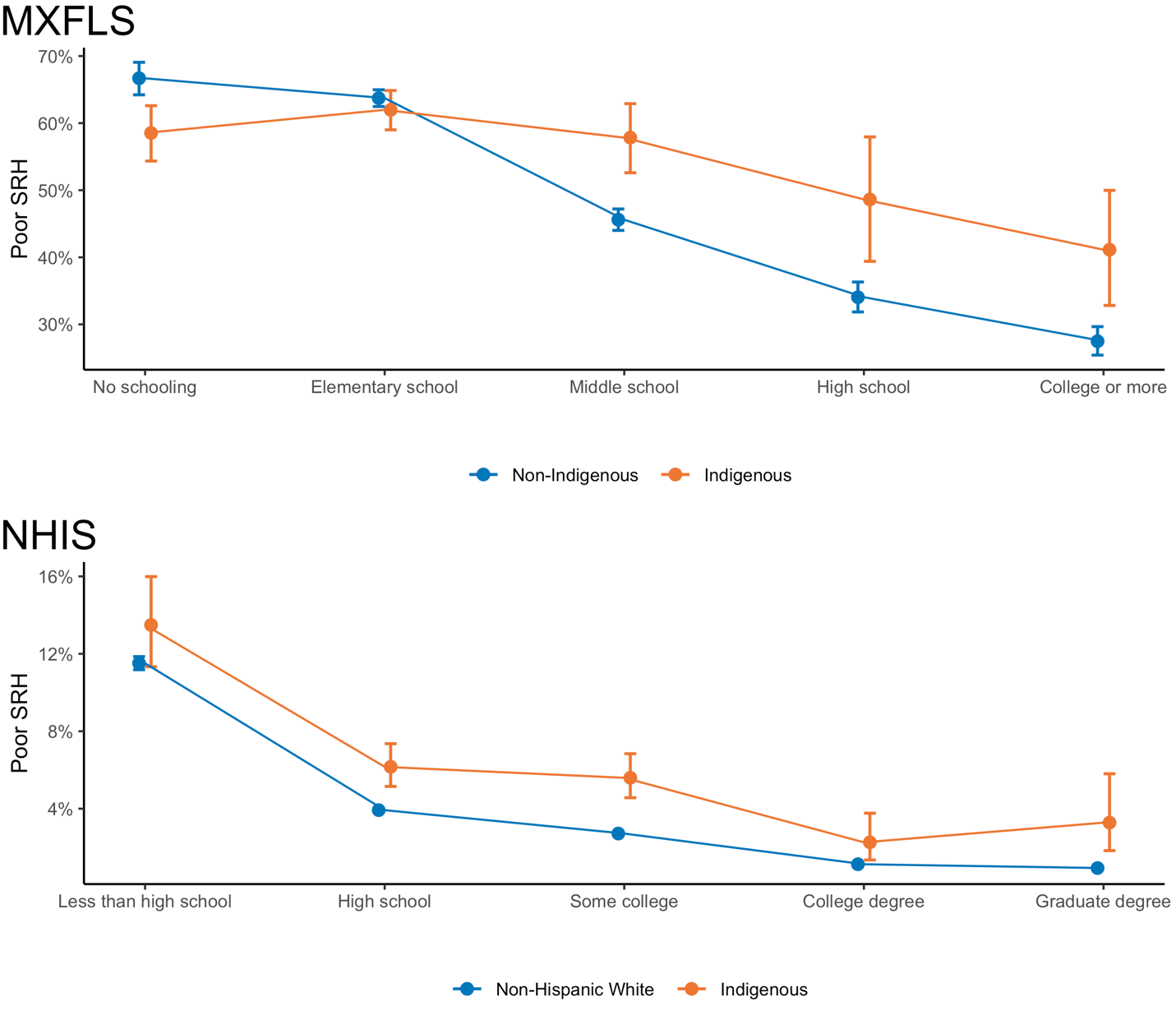 |
| *Note: Data comes from the Mexican Family Life Survey (top) and National Health Interview Survey, 2000-2018 (bottom). Figures depict predicted probability fair/poor self-rated health based on logistic regression models with interactions between educational attainment and a categorical variable comparing Indigenous and non-Indigenous status.* |
